# Use of Autologous Blood Components in Lymphedema Treatment: A Systematic Review

**DOI:** 10.7759/cureus.5638

**Published:** 2019-09-12

**Authors:** Antonio J Forte, Daniel Boczar, Maria T Huayllani, Sanjay Bagaria, Sarah A McLaughlin

**Affiliations:** 1 Plastic Surgery, Mayo Clinic Florida - Robert D. and Patricia E. Kern Center for the Science of Health Care Delivery, Jacksonville, USA; 2 Surgery, Mayo Clinic Florida - Robert D. and Patricia E. Kern Center for the Science of Health Care Delivery, Jacksonville, USA

**Keywords:** lymphedema, chronic lymphedema, targeted therapy, blood components

## Abstract

The main benefit of autologous therapies is its easier obtention and relatively lower concerns regarding ethical implications and patient safety. We conducted a systematic review of publications assessing the potential use of blood components (lymphocytes, red blood cells (RBCs), platelet-rich plasma (PRP)) as targeted therapy in the treatment of lymphedema. We hypothesized that blood components could be used as targeted therapy in the lymphedema treatment. We also conducted a comprehensive, systematic review of the published literature on the use of blood components as targeted therapies in the treatment of lymphedema using the PubMed database. Eligibility criteria excluded papers that aimed to investigate the correlation of inflammatory cells and the physio-pathogenesis of lymphedema. Abstracts, presentations, reviews, and meta-analyses were also excluded. From the 338 potential papers found in the literature, 11 studies fulfilled the eligibility criteria. Different types of targeted therapies were proposed, but the majority of papers investigated the potential use of lymphocytes (9/11). The use of PRP was investigated in two papers and the use of RBCs in one paper. Interestingly, six out of 11 studies were done on patients with lymphedema, but the most recent was published in 1999. The remaining publications were experimental studies on dogs, rats, or in vitro. The publications demonstrated positive outcomes for the delivery of lymphocytes and PRP in lymphedema treatment. Lymphocyte was the most common blood component investigated. Even though more than half of the papers found were conducted on patients, we noticed a scientific gap of more than 20 years on the topic.

## Introduction and background

It is estimated that one in every six patients with solid cancer undergoing treatment will develop secondary lymphedema. Only in the United States, five to six million people are affected by this condition that is still considered incurable [1]. The physiopathology of lymphedema has been studied, validating the important role of tissue inflammation and fibrosis, which explains why most patients present clinical findings months after the primary lymphatic damage [2-4].

Searching for therapies to alleviate or potentially cure lymphedema, authors have proposed targeted therapies to modulate tissue inflammation, fibrosis, and lymphangiogenesis. These therapies could be clustered into two main groups: (1) Non-autologous therapies, that needed to be produced artificially (e.g. growth factors, cytokines, viral vectors, medications, etc.) [5-6]; or (2) Autologous therapies that could potentially be obtained from own patient’s body (stem cells and blood components such as lymphocytes, and platelet-rich plasma). Nonetheless, the clinical application of such therapies raises numerous concerns such as the potential risk of metastasis in cancer patients [6].

Compared to non-autologous therapies, the main benefit of autologous therapies is its easy obtention and relatively lower concerns regarding ethical implications and patient safety [7]. Studies conducted on adipose-derived stem cells in the lymphedema treatment have demonstrated its capacity to promote lymphangiogenesis in vitro and in vivo [8-10]. Nonetheless, blood components also have a high physiological potential in the lymphedema treatment since lymphangiogenic cytokines could be found in blood’s platelets, and lymphocytes are well-known regulators of inflammation in the lymphedema tissue [11-14].

Currently, lymphedema treatments are still passible of unpredictable outcomes, demonstrating the urge for studies about targeted therapies passible of clinical translation [15]. Therefore, we conducted a systematic review of publications assessing the potential use of blood components (lymphocytes, erythrocytes, platelet-rich plasma) as targeted therapy in the treatment of lymphedema. We hypothesized that blood components could be used as a targeted therapy in the lymphedema treatment.

## Review

Methods

Search Strategy

**Figure 1:**
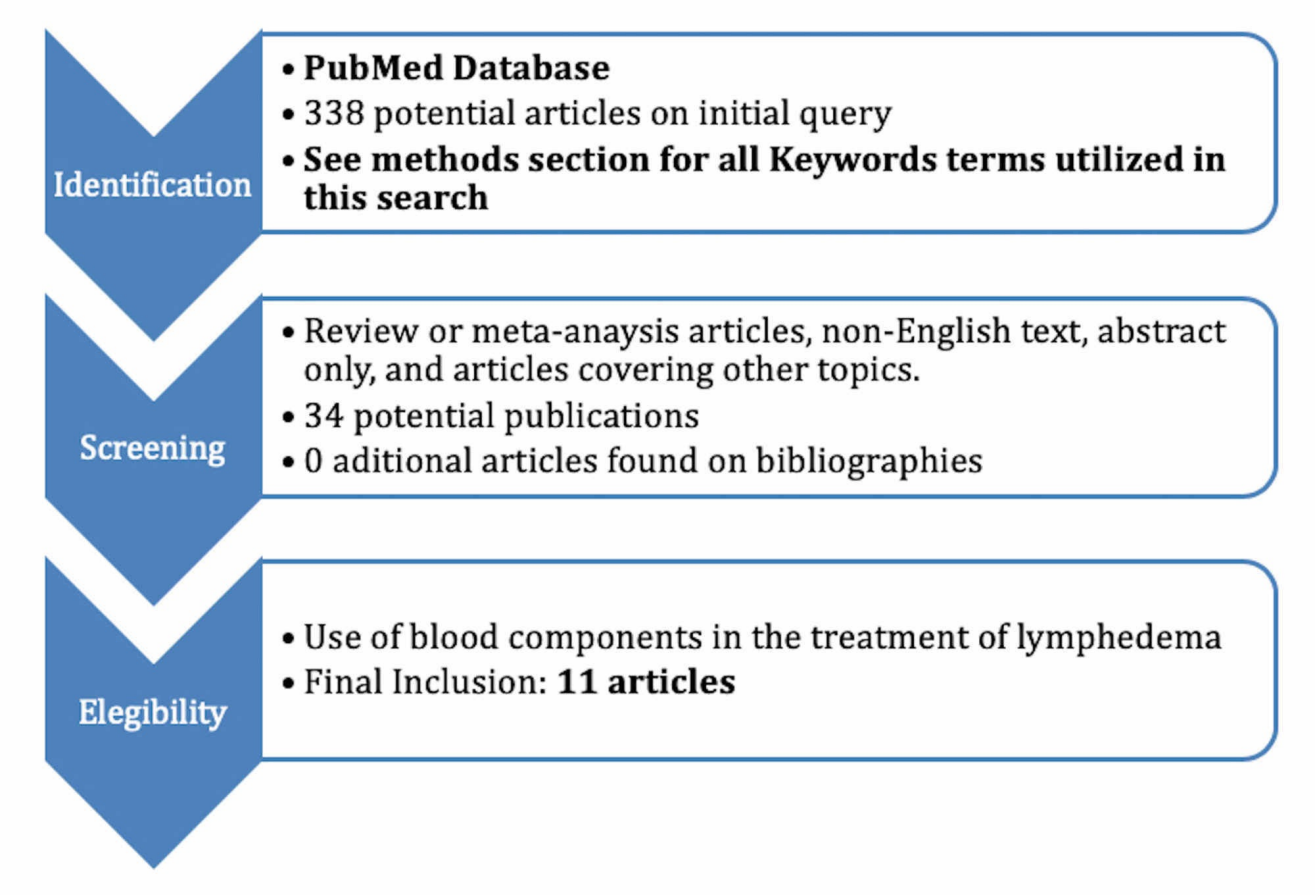
Flowchart of Article Identification and Final Selection

Two reviewers (D.B., M.T.) conducted independent searches using the PubMed database without timeframe limitations, initially through title and abstract screens and then by full-text review. Disagreements regarding article identification and final selection for the inclusion of the literature were resolved by another reviewer (A.J.F). The search was done using the following keywords: (((((Platelet-rich plasma) OR PRP)) OR ((autologous lymphocyte) OR lymphocyte)) OR ((Erythrocytes) OR Red blood cells)) AND ((Lymphedema) OR Breast cancer lymphedema). The bibliographies of the studies that fulfilled the study eligibility criteria were also examined, looking for articles not present in our initial search. This study followed the guidelines outlined in the preferred reporting items for systematic reviews and meta-analyses (PRISMA) flowchart in Figure 1. 

Selection Criteria

Eligibility criteria included studies reporting data from the use of blood components as targeted therapies in the treatment of lymphedema. Therefore, we excluded papers that aimed to investigate the correlation of inflammatory cells and the physio-pathogenesis of lymphedema. Abstracts, presentations, reviews, and meta-analyses were also excluded.

Data Extraction and Processing

Extracted data included the year of study, country, type of study, model used for experiments, blood components, and therapy delivery. Data extraction from articles, tables, and figures was performed by two reviewers (D.B., M.T.), with the accuracy of data entry confirmed by an additional reviewer (A.J.F).

Results

Studies Description

From 338 potential papers found in the literature, 11 studies fulfilled the study eligibility criteria (Figure 1, Table 1). The potential use of blood components as targeted therapy in the lymphedema treatment was described by groups from different countries but most of them (7/11) were from Japan. The first publication on the topic was also from Japan - Katoh et al. in 1984 [16]. Different types of targeted therapies using blood components in the lymphedema treatment were proposed but the majority of papers investigated the potential use of lymphocytes (9/11). The use of PRP was investigated in two papers and the use of RBCs in one paper. Interestingly, six out of 11 studies were done on patients with lymphedema. The remaining publications were experimental studies in dogs, rats, or in vitro.

**Table 1:** Summary of Studies Investigating Blood Components as Targeted Therapies in Lymphedema Treatment RBC, Red blood cells; PRP, Platelet-rich plasma

Author	Year	Country	Type of Study	Lymphedema Model	Component	Delivery
Katoh et al. [16]	1984	Japan	Serie of cases	Patient	Lymphocyte	Intra-arterial
Yoshizumi et al. [20]	1992	Japan	Case report	Patient	Lymphocyte	Intra-arterial
Egawa et al. [21]	1993	Japan	Case report	Patient	Lymphocyte	Intra-arterial
Harada et al. [17]	1994	Japan	Case series	Patient	Lymphocyte	Intra-arterial
Knight et al. [22]	1994	Australia	Animal study	Canine	Lymphocyte	Intra-arterial
Nagata et al. [18]	1994	Japan	Animal study	Patient	Lymphocyte	Intra-arterial
Ogawa et al. [19]	1999	Japan	Case series	Patient	Lymphocyte	Intra-arterial
Hadamitzky et al. [26]	2009	Germany	Animal study	Rat	RBC or PRP	Intradermally
Ackermann et al. [25]	2015	Germany	Animal study	Rat	PRP	Subcutaneously
Gousopoulos et al. [23]	2016	Switzerland	Animal study	Rat	Lymphocyte	Intravenous
Itoh et al. [24]	2016	Japan	In vitro	-	Lymphocyte	-

Lymphocytes

Delivery of lymphocytes was assessed in nine publications, all agreeing that its administration promotes positive results on lymphedema. Authors conducted studies on patients injecting lymphocytes in arteries proximal to the affected limbs. Katoh et al. described that five out of seven patients demonstrated clinical reduction of the lymphedema, evident even following the first injection of the lymphocytes. Initially, the lymphocytes were obtained from healthy donors, but after one of their patients developed a graft-vs-host reaction, they started injecting lymphocytes from the patients' own blood [16]. Harada et al. observed improvement in lymphedema in three out of five patients with unilateral leg lymphedema, which was quantitatively measured by measuring the T2 relaxation time on magnetic resonance imaging (MRI) [17]. Nagata et al. conducted a study on 13 patients with refractory lymphedema. They pointed out a softening of the affected limb in all patients. Moreover, in their cohort, patients experienced a reduction in the size of the limb (mean 64%) and a decrease in ache and sensation of heat in the affected limb. Reduction in size was maintained in nine patients for three months [18].

Ogawa et al. conducted a study on 46 patients with unilateral lymphedema, demonstrating that lymphocyte injection associated with compression methods alleviated lymphedema in 74% (34/46 patients) and promoted an expressive reduction of lymphedema in 37% (17/46 patients) [19].To understand the mechanism in which lymphocyte injection could improve lymphedema, they studied the expression of L-selectin (lymphocyte cell adhesion molecule) in five cases of their cohort. Increased expression of L-selectin was found in the lymphocytes collected from peripheral blood, and they postulated that lymphocytes positive for L-selectin and negative for CD continued in the affected swollen limb, regulating local immune responses [19]. Interestingly, the other two authors shed light on the capacity of the injected lymphocytes to remain in the tissue after the injection of lymphocytes in lymphedema patients. Yoshimuzi et al. pointed out the presence of specific lymphocyte markers in the lymphedema tissue, and Egawa et al. pointed out changes in the protein components of the lymphedema fluid [20-21].

The use of lymphocytes in lymphedema treatment was also supported by experimental studies. Knight et al, in a study on a canine model where they injected lymphocytes into the proximal artery of the affected limb, pointed out a reduction of 69% in the edema of the affected limb as compared to the normal limb, which served as control [22]. Moreover, they noticed a reduction in fibrosis (skin thickness and hydroxyproline), protein concentration, and water content. They postulated that a lymphocyte injection possibly activated macrophages, promoting proteolysis [22]. Gousopoulos et al. performed an experiment on rats injecting T-regulatory cells, where they noticed that the expansion of those cells reduced lymphedema through a reduction of edema, fibrosis, inflammation, and improvement of lymphatic drainage [23]. Itoh et al. performed an In vitro experiment in which they induced human T-cell lymphocytes to become pluripotent stem cells through the exogenous expression of four factors (OCT3/4, SOX2, cMYC, and KLF4) using viral vectors. They postulated that those stem cells derivated from lymphocytes could be useful for the in vitro development of lymphatic vessels [24].

Plasma-rich Protein and Red Blood Cells

Two authors pointed out good results related to the use of PRP in lymphedema treatment. Ackermann et al. conducted an experimental study on rats injecting fresh human blood PRP, adipose-derived stem cells, or saline into wounds [25]. Compared to the control, PRP injection promoted faster wound healing and increased epithelialization. Moreover, they were able to demonstrate that PRP increased lymphangiogenesis and lymphatic vessel density. Rats that received a PRP injection had a significant decrease in lymphedema at Days7 and 14 of their experiment [25]. Hadamitzky et al. conducted an experimental study on rats that underwent lymph node transplantation, injecting PRP or sheep RBCs intradermally. They noticed that PRP injection induced an improvement in the regeneration and preservation rate of the transplanted lymph nodes. However, the RBC injection did not demonstrate positive results in their experiments on lymphedema [26].

Discussion

In this systematic literature review, we have shown that different blood components were proposed as targeted therapies in the lymphedema treatment. Despite the limit amount of studies published on the topic and the presence of several limitations in clinical studies, such as the low number of patients, the use of lymphocytes and PRP, seems to be promising. The literature on lymphedema pathogenesis and treatment has been increasing considerably over the years. Interestingly, the last clinical study done using blood components was published in 1999, representing a scientifical gap of 20 years. To our knowledge, this study is the first systematic literature review assessing the potential use of blood components as targeted therapies in lymphedema treatment.

The idea that using a lymphocyte injection in lymphedema treatment originated by findings on studies about immuno-adaptive treatment for breast cancer [27-28]. Interestingly, the studies included in this systematic review pointed out the benefits of using lymphocyte therapy, including clinical improvement of patients with lymphedema refractory to conventional treatment [18].

The idea of using PRP was originated by the physiologic characteristics of the platelets, which are considered pools of potent growth factors with angiogenic and lymphangiogenic properties [11]. Their granules contain growth factors, such as platelet-derived growth factor, platelet-derived epidermal growth factor, insulin growth factor, platelet-derived angiogenesis factor, and growth factors, of the vascular endothelial growth factor family [11].

We do recognize the presence of several limitations to our study, common to systematic reviews, such as the potential for bias in interpreting the data collected from the studies. Moreover, we only included in this review papers that were published in the English language. However, we believe that this study summarized valuable data, particularly about the potential use of blood components as a targeted therapy in lymphedema treatment, which can guide future studies to advance the field.

## Conclusions

The pooled publications about the potential use of blood components as targeted therapy in lymphedema treatment demonstrated positive outcomes for the delivery of lymphocytes and PRP. Lymphocyte was the most common blood component investigated, followed by PRP. To date, only one study used RBCs, but no positive outcomes were observed. Even though more than half of the papers found were conducted on patients, the most recent clinical study was published in 1999, evidencing a gap of 20 years, which evokes the necessity of further clinical studies on the topic.
